# The complete mitochondrial genome of *Bauhinia variegata* (Leguminosae)

**DOI:** 10.1080/23802359.2024.2305712

**Published:** 2024-01-18

**Authors:** Chenyu Sun, Yong Chen, Danjing Zheng, Yan Zhong, Shukai Luo, Shiyuan Meng, Lei Qian, Dan Wei, Ying Liu, Seping Dai, Renchao Zhou

**Affiliations:** a School of Life Sciences, State Key Laboratory of Biocontrol and Guangdong Provincial Key Laboratory of Plant Resources, Sun Yat-sen University, Guangzhou, China; b Guangzhou Institute of Forestry and Landscape Architecture, Guangzhou Collaborative Innovation Center on Science-Tech of Ecology and Landscape, Guangzhou, China; c Guangdong Academy of Forestry, Guangdong Provincial Key Laboratory of Silviculture, Protection and Utilization, Guangzhou, China

**Keywords:** mitochondrial genome, gene annotation, *Bauhinia*, phylogenetic analysis

## Abstract

The mitogenome of *Bauhinia variegate* was assembled and characterized in this study. The mitogenome size was 437,271 bp, and its GC content was 45.5%. 36 protein-coding genes, 17 tRNAs and 3 rRNAs were annotated in the mitogenome. A total of 12 MTPTs, ranging from 71 bp to 3562 bp, were identified in the mitogenome and covered 1.46% (6373 bp) of the mitogenome. Phylogenetic analysis of 15 species of Leguminosae based on 23 core protein-coding genes showed that *B. variegata* was sister to *Tylosema esculentum*, another member from the subfamily Cercidoideae. The mitogenome of *B. variegata* provides a valuable genetic resource for further phylogenetic studies of this family.

## Introduction

Leguminosae consists of six well-supported subfamilies, i.e. Papilionoideae, Caesalpinioideae, Detarioideae, Cercidoideae, Dialioideae and Duparquetioideae (LPWG 2017). Within the subfamily Cercidoideae, *Bauhinia* is the largest genus and comprises 380 species distributed in the pantropical regions (LPWG 2017). With large flowers and long flowering period, species of *Bauhinia* are well-known ornamental trees of the legume family. Three species, *B. variegate*, *B. purpurea* and their hybrid *B*. × *blakeana* (Carol et al. 2005; Mak et al. 2008), are widely cultivated in South China and other tropical and subtropical regions in the world.

Genomic data are important genetic resources for resolving phylogenetic relationships and dissecting genes underlying key traits. The nuclear genome of *Bauhinia variegata* (Zhong et al. 2022) and chloroplast genomes of four species of *Bauhinia* (Wang et al. 2018; Gu et al. 2019, 2020; Xiao et al. 2022) have been characterized. However, no mitogenomes have been sequenced for *Bauhinia* to date. Here, we assembled the mitogenome of *B. variegata* using Illumina sequencing reads and aimed to determine the phylogenetic position of *Bauhinia* based on phylogenetic analysis of mitogenome sequence data.

## Materials and methods

**Figure 1.**
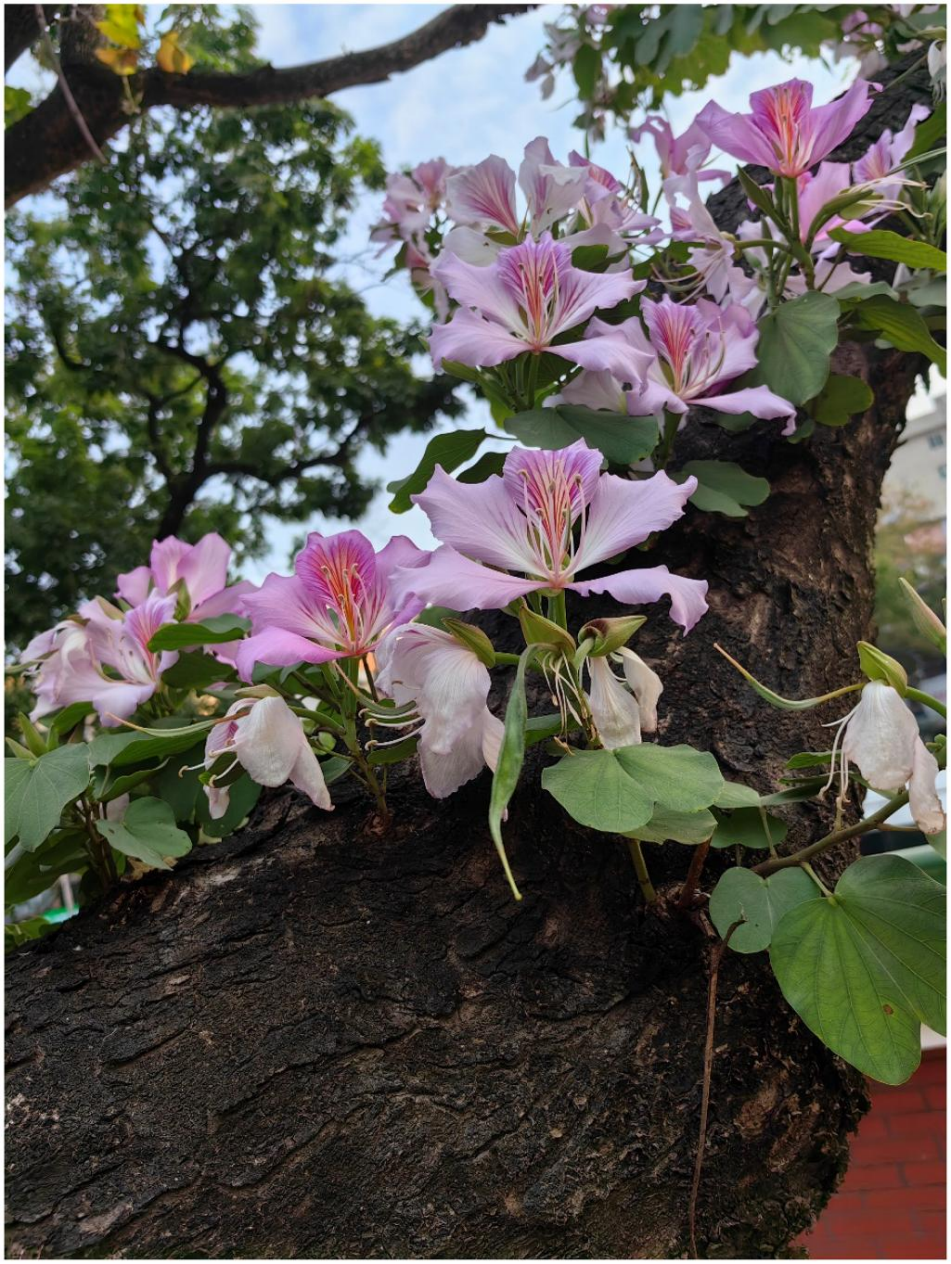
An individual of *Bauhinia variegata* showing its dark brownish trunk, two-lobed green leaves, pink flowers and young, green fruits. This individual was collected from the campus of Sun Yat-sen University, Guangzhou, China, and photographed by Renchao Zhou.

*Bauhinia variegata* is not an endangered or protected species, so no permissions are required for sampling. Fresh young leaves of a *B. variegata* individual (Figure 1) were collected from the campus of Sun Yat-sen University (N113°18'8″, E23°5'24″), Guangzhou, Guangdong, China. A voucher specimen (SYS-2021-11-20) was identified by Dr. Yong Chen and deposited at Sun Yat-sen University Herbarium (SYS) under the charge of Prof. Wenbo Liao (lsslwb@mail.sysu.edu.cn). Illumina sequencing of the *B. variegata* individual was done on an Illumina NovaSeq platform (Zhong et al. 2022), and 48.8 Gb paired-end reads of 150 bp were generated. Trimmomatic v0.39 (Bolger et al. 2014) was used to filter the Illumina reads with default parameters. Clean reads were used to assemble the mitogenome using GetOrganelle v1.6.4 (Jin et al. 2020) with parameters -R set to 15 and -k set to 127. The original assemblies were visualized with Bandage (Wick et al. 2015), and mitochondrial contigs were chosen based on their depths and high sequence similarity to the mitogenome of *Cercis canadensis* (NCBI accession number: MN017226.1). The mitochondrial contigs could be arranged into a circular molecule in Bandage and exported as the mitogenome sequence of *B. variegata*. To clarify the accuracy of the assembly, we further mapped our clean reads back to the assembled mitogenome using BWA-mem (Li and Durbin 2010) to assess the depth of coverage (Figure S1). Mitochondrial protein-coding genes and rRNAs were annotated using Geseq (Tillich et al. 2017) with the mitogenome of *Cercis canadensis* as a reference. Annotation of tRNAs was executed by tRNAscan-SE v2.0 (Lowe and Chan 2016) with the ‘organelle’ mode. Gene map of the mitogenome and maps of genes containing *cis*- and *trans*-spliced introns were drawn by PMGmap (Zhang et al. 2023).

To identify the mitochondrial sequences of plastid origin (MTPTs), the chloroplast genome sequence of *B. variegata* (Gu et al. 2020) were used as a query to search the mitogenome sequence using Blastn (Chen et al. 2015) with default parameters. The identified MTPTs were also annotated using Geseq. The MTPTs in the mitogenome and their counterparts in the chloroplast genome were visualized using TBtools (Chen et al. 2023).

To infer the phylogenetic position of *B. variegata* in Leguminosae, sequences of 23 ‘core’ mitochondrial protein-coding genes (*cox2* was excluded because it is missing in *Vigna radiata*) of 15 species in this family (including *B. variegata*) were used for phylogenetic reconstruction. *Malus domestica* from Rosaceae was used as the outgroup. All the mitochondrial protein gene sequences of these species except *B. variegata* were downloaded from GenBank (see Table S1 for details). Sequences were aligned with MAFFT v7.307 (Katoh and Standley 2013), and a maximum likelihood (ML) tree was constructed based on the aligned sequences using RAxML v8.2.12 (Stamatakis 2014) with a GTRGAMMA substitution model and 1000 bootstrap replicates.

## Results

**Figure 2.**
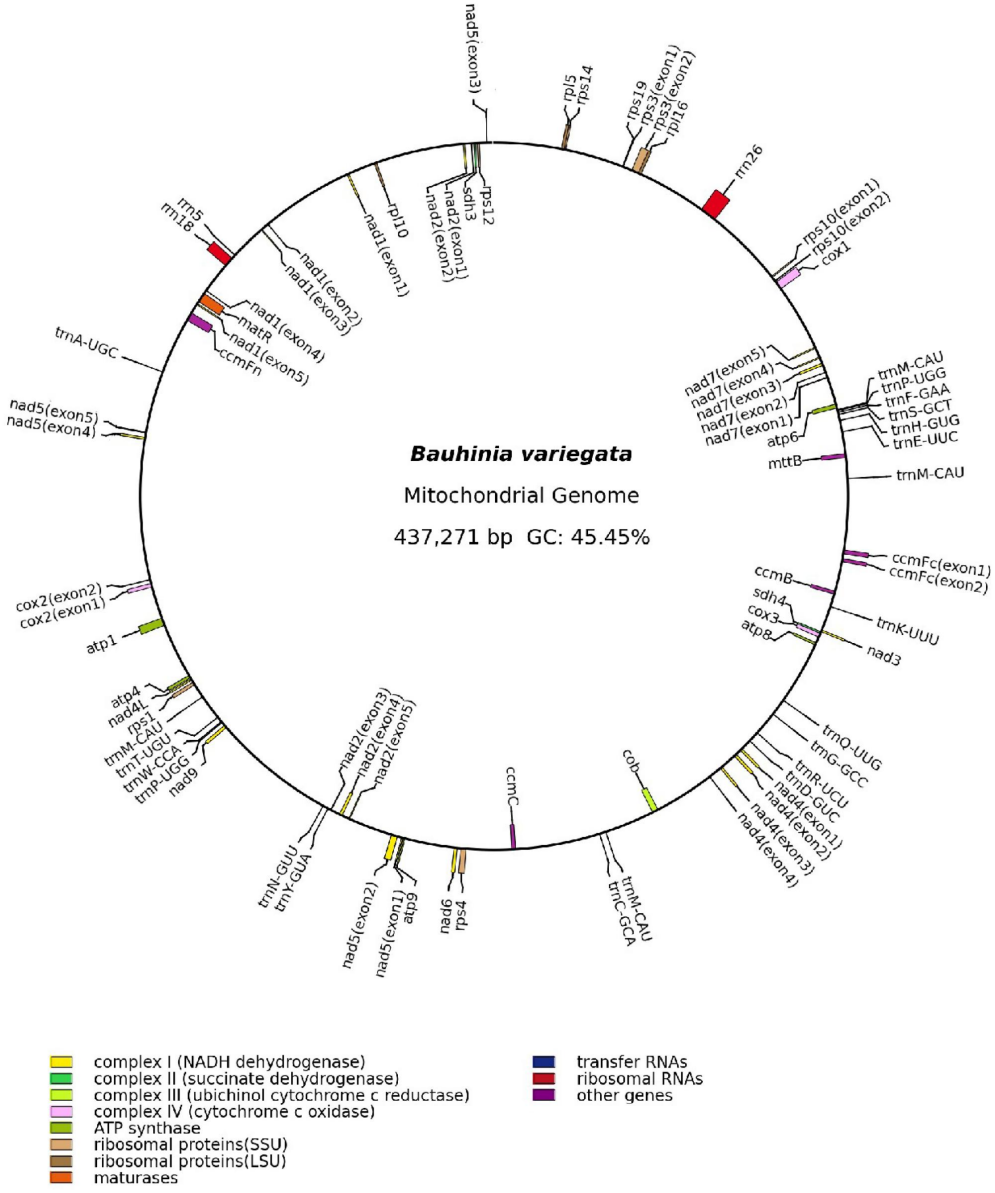
The mitogenome map of *Bauhinia variegata*. Genes shown outside and inside the outer circle are transcribed clockwise and counterclockwise, respectively.

**Figure 3.**
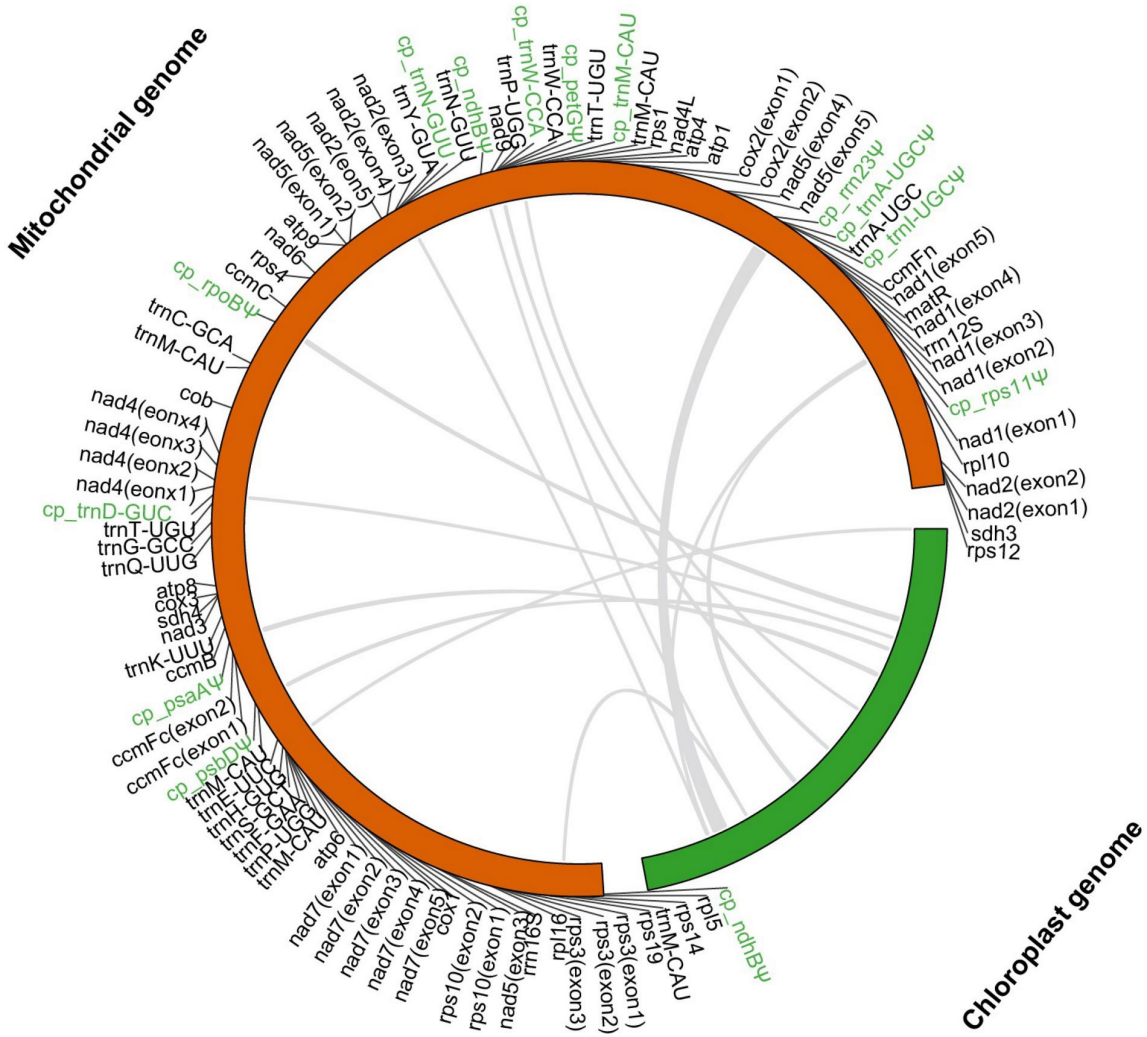
Mitochondrial sequences of plastid origin (MTPTs) in the mitogenome of *Bauhinia variegata*. Chloroplast genome (excluding one IR) is shown in green and mitochondrial genome is colored in orange. Grey lines within the circle indicate transferred regions from the chloroplast genome. Genes or pseudogenes (with a symbol ‘Ψ’) transferred from the chloroplast genome are marked in green.

The assembled mitogenome of *B. variegata* was a single master circle of 437,271 bp, and its overall GC content was 45.5%. The sequencing depth was relatively even across the mitogenome, with an average depth of 5791.9 × (Figure S1), indicating the continuity of the mitogenome assembly. 36 unique protein-coding genes, 17 unique tRNA genes, and 3 unique rRNA genes were annotated in the mitogenome of *B. variegate* (Figure 2). The total length of protein-coding genes was 31,129 bp, covering 7.1% of the genome. The 36 protein-coding genes contained 24 core genes that are present in the common ancestor of seed plants (Mower et al. 2012) and 12 genes variably present in extant seed plants. In this mitogenome, there are 23 introns, 18 are *cis*-spliced introns (intron1 and intron2 of *nad1*, intron1, intron3 and intron4 of *nad2*, intron1, intron2 and intron3 of *nad4*, intron1 and intron4 of *nad5*, intron1, intron2, intron3 and intron4 of *nad7*, intron1 of *ccmFc*, intron1 of *cox2*, intron1 of *rps10* and intron1 of *rps3*) and 5 are *trans*-spliced introns (intron3 and intron4 of *nad1*, intron2 of *nad2*, intron2 and intron3 of *nad5*) (Figure S2). A total of 12 MTPTs, ranging from 71 bp to 3562 bp, were identified in the mitogenome of *B. variegate*, and they cover 1.46% (6373 bp) of the mitogenome (Figure 3).

**Figure 4.**
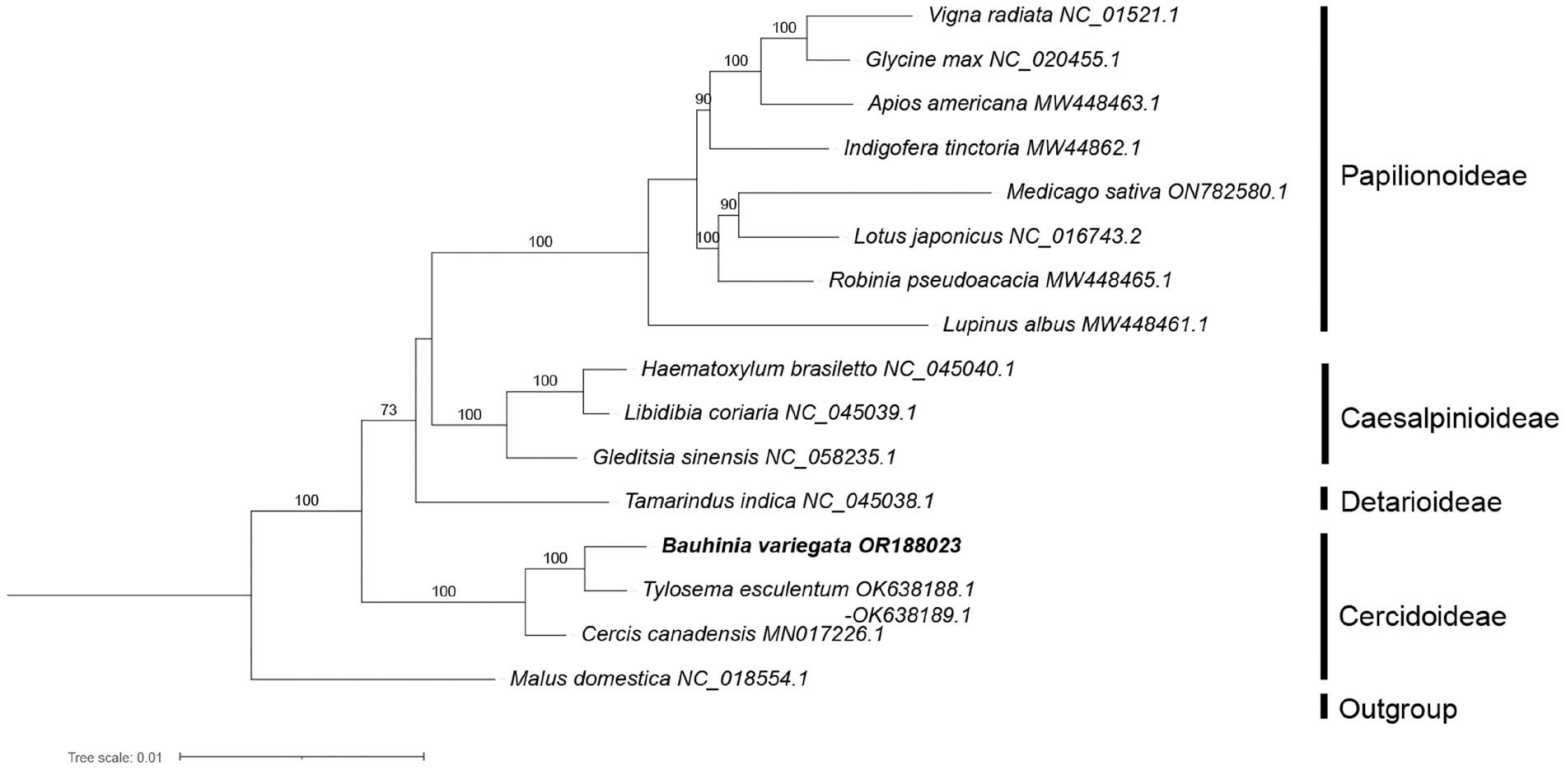
Maximum likelihood tree of 15 species from the Leguminosae family based on 23 concentrated mitochondrial genes, with *Malus domestica* from Rosaceae as the outgroup. Bootstrap support values, based on 1000 replicates, are shown on each node. For each species, GenBank accession number of its mitogenome sequence is shown, following the species name. See Table S1 for citation details of the sequences used.

Based on the concatenated 23 core protein-coding genes (*atp1, atp4, atp6, atp8, atp9, ccmB, ccmC, ccmFc, ccmFn, cob, cox1, cox3, matR, mttB, nad1, nad2, nad3, nad4, nad4L, nad5, nad6, nad7* and *nad9*), phylogenetic analysis showed that the 15 species of Leguminosae were divided into four clades, corresponding to the four subfamilies of Leguminosae, and that *B. variegata* aligned as a sister group to *Tylosema esculentum*, another member from the subfamily Cercidoideae, with 100% bootstrap support (Figure 4).

## Discussion and conclusion

Leguminosae is the third largest family in angiosperms (LPWG 2017). With many species being of great economical and agronomical significance, phylogeny of this family has attracted much attention. The six well-supported subfamilies were resolved based on phylogenetic analysis of chloroplast *matK* sequences (LPWG 2017). Later phylogenetic analysis based on chloroplast genome sequences or many nuclear genes produced almost identical results about the six subfamilies (Koenen et al. 2020; Zhang et al. 2020). However, the relationships between these subfamilies and genera among the subfamilies are not well resolved. The latest phylogenomics study using transcriptomic and genomic data resolved the relationships among the subfamilies and found that the clade comprising subfamilies Cercidoideae and Detarioideae was sister to the remaining legumes (Zhao et al. 2021).

Mitogenome sequences are a valuable source for resolving phylogenetic relationships of plants. However, mitogenome sequences have not been used in phylogenetic analyses of Leguminosae. In this study, the phylogeny of four subfamilies of Leguminosae with available mitogenomic sequences was constructed and the topology was consistent with previous studies from chloroplast and nuclear genome sequences, suggesting mitogenome sequences might be very valuable in phylogenetic studies in Leguminosae. So far, only four subfamilies of Leguminosae have available mitogenome sequences. Mitogenome sequences from two other subfamilies, Dialioideae and Duparquetioideae, are needed to reconstruct the family-level phylogeny. The mitogenome of *B. variegata* characterized in this study will benefit phylogenetic study of this family.

## Ethical approval

No ethical approval is needed for this study. *Bauhinia variegata* is not an endangered or protected species.

## Supplementary Material

Supplemental Material

## Data Availability

The data that support the findings of this study are openly available in NCBI (https://www.ncbi.nlm.nih.gov/) under the accession number OR188023. The associated BioProject, SRA and Bio-Sample numbers are PRJNA801801, SRR24980125 and SAMN25391341.
