# Supplementary material for: The complete mitochondrial genome of Bauhinia variegata (Leguminosae)
Source: Mitochondrial DNA B Resour. 2024 Jan 18;9(1):128–32. doi: 10.1080/23802359.2024.2305712 (PMC10802806; doi:10.1080/23802359.2024.2305712)
Supplement: Supplemental Material [file TMDN_A_2305712_SM0091.docx]

**Supplementary Materials**

**Table S1** Species and their GenBank accession numbers used in this study

| **Species** | **Accession No.** | **Source** |
| --- | --- | --- |
| *Bauhinia variegata* | OR188023 | This study |
| *Cercis canadensis* | MN017226.1 | Choi et al. 2018 |
| *Tylosema esculentum* | OK638188.1  OK638189.1 | Li and Cullis 2021 |
| *Tamarindus indica* | NC_045038.1 | Choi et al. 2018 |
| *Robinia pseudoacacia* | MW448465.1 | Choi et al. 2018 |
| *Apios americana* | MW448463.1 | Choi et al. 2021 |
| *Medicago sativa* | ON782580.1 | unpublished |
| *Gleditsia sinensis* | NC_058235.1 | unpublished |
| *Glycine max* | NC_020455.1 | Chang et al. 2013 |
| *Haematoxylum brasiletto* | NC_045040.1 | Choi et al. 2018 |
| *Vigna radiata* | NC_015121.1 | Alverson et al. 2011 |
| *Indigofera tinctoria* | MW448462.1 | Choi et al. 2021 |
| *Libidibia coriaria* | NC_045039.1 | Choi et al. 2018 |
| *Lotus japonicus* | NC_016743.2 | Kazakoff et al. 2012 |
| *Lupinus albus* | MW448461.1 | Choi et al. 2021 |
| *Malus* *domestica* | NC_018554.1 | Goremykin et al. 2021 |

Alverson AJ, Zhuo S, Rice DW, Sloan DB, Palmer JD. 2011. The mitochondrial genome of the legume *Vigna radiata* and the analysis of recombination across short mitochondrial repeats. PLoS One. 6(1): e16404.

Chang S, Wang Y, Lu J, Gai J, Li J, Chu P, Guan R, Zhao T. 2013. The mitochondrial genome of soybean reveals complex genome structures and gene evolution at intercellular and phylogenetic levels. PLoS One. 8(2): e56502.

Choi IS, Schwarz EN, Ruhlman TA, Khiyami MA, Sabir JSM, Hajarah NH, Sabir MJ, Rabah SO, Jansen RK. 2018. Fluctuations in Fabaceae mitochondrial genome size and content are both ancient and recent. BMC Plant Biol. 19(1):448.

Choi IS, Wojciechowski MF, Ruhlman TA, Jansen RK. 2021. In and out: Evolution of viral sequences in the mitochondrial genomes of legumes (Fabaceae). Mol Phylogenet Evol. 163:107236.

Goremykin VV, Lockhart PJ, Viola R, Velasco R. 2012. The mitochondrial genome of *Malus domestica* and the import-driven hypothesis of mitochondrial genome expansion in seed plants. Plant J. 71(4):615-26.

Kazakoff SH, Imelfort M, Edwards D, Koehorst J, Biswas B, Batley J, Scott PT, Gresshoff PM. 2012. Capturing the biofuel wellhead and powerhouse: the chloroplast and mitochondrial genomes of the leguminous feedstock tree *Pongamia pinnata*. PLoS One. 7(12): e51687.

Li J, Cullis C. 2021. The multipartite mitochondrial genome of Marama (*Tylosema esculentum*). Front Plant Sci 12: 787443

**
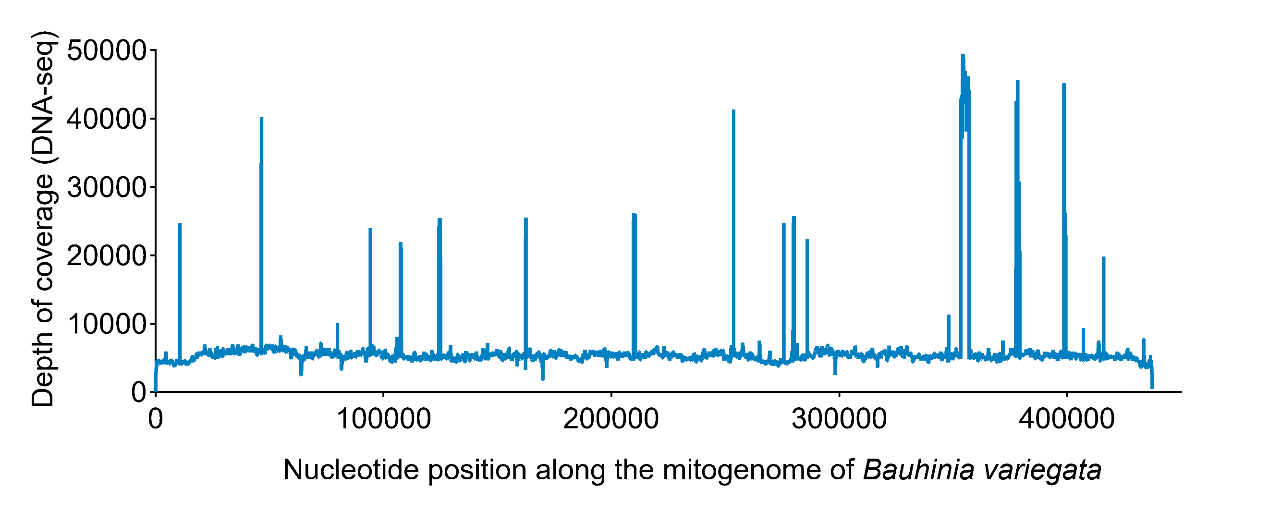
**

**Figure S1** Depth of coverage along the mitogenome of *Bauhinia variegata*. Superhigh depth of coverage on multiple positions in this figure represent mitochondrial sequences of plastid origin (MTPTs).

**
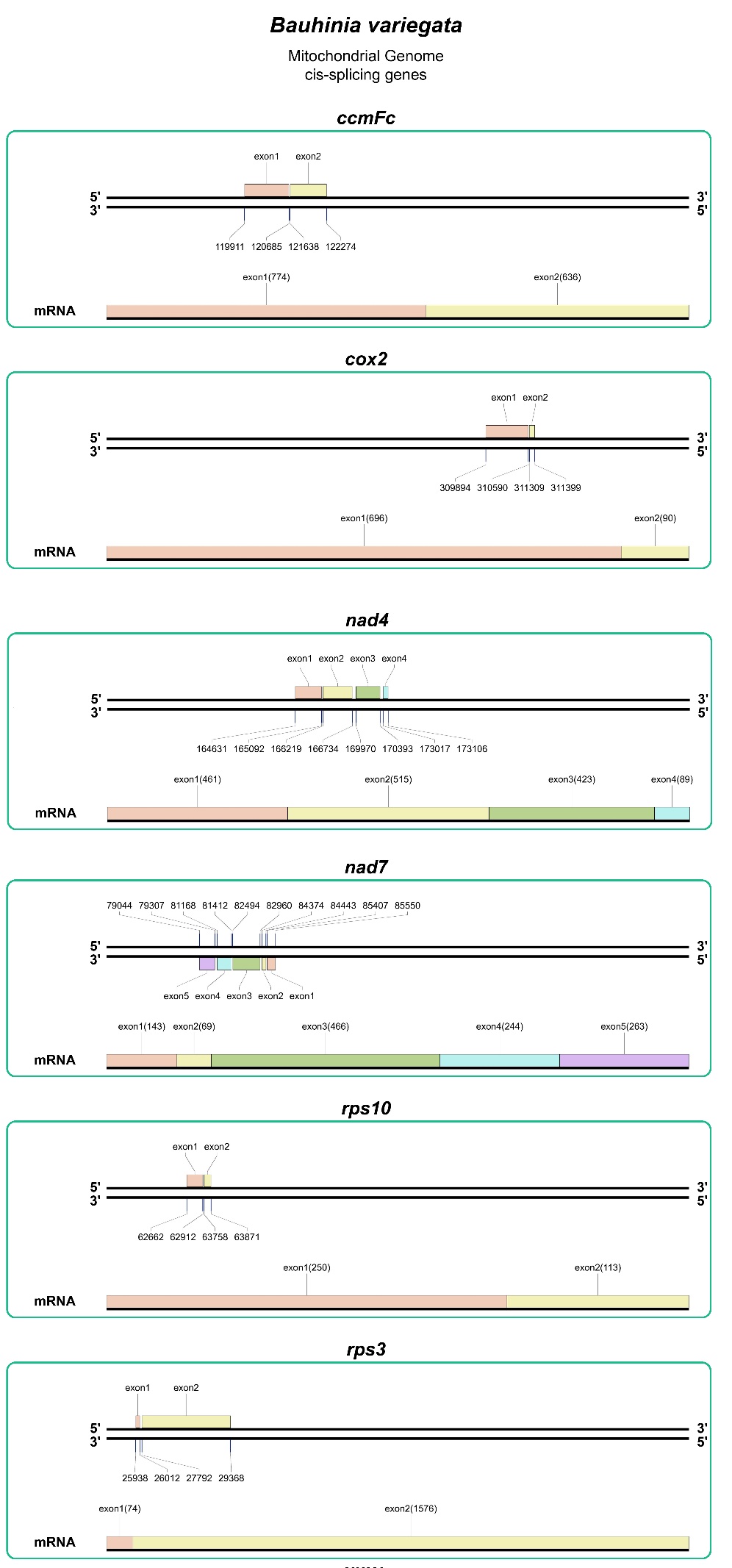
**

A

**
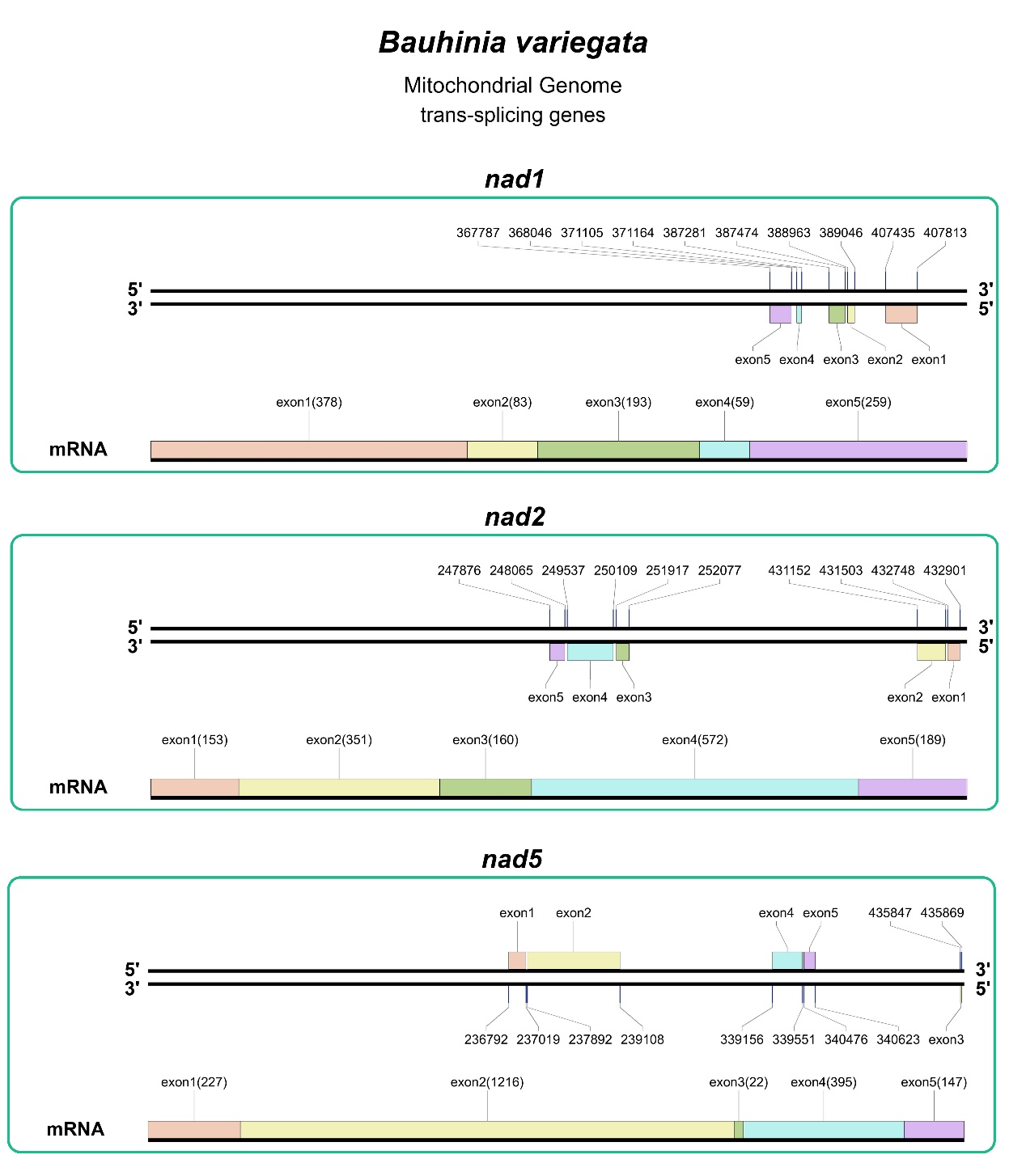
**

B

**Figure S2** Maps of *cis*- and *trans*-spliced genes in the mitogenome of *Bauhinia variegata*. A and B display genes containing only *cis*-spliced introns and genes containing *trans*-spliced introns, respectively. This figure was drawn by PMGmap.
